# Citizen science helps to raise awareness about gut microbiome health in people at risk of developing non-communicable diseases

**DOI:** 10.1080/19490976.2023.2241207

**Published:** 2023-08-02

**Authors:** Silvia Garcia, Sheyla Ordoñez, Victor Manuel López-Molina, Blanca Lacruz-Pleguezuelos, Enrique Carrillo de Santa Pau, Laura Judith Marcos-Zambrano

**Affiliations:** Computational Biology Group, Precision Nutrition and Cancer Research Program, IMDEA Food Institute, Madrid, Spain

**Keywords:** 16S rRNA gene sequencing, co-occurrence networks, keystone taxa, participatory action research, citizen science, microbiota, photovoice

## Abstract

Citizens lack knowledge about the impact of gut microbiota on health and how lifestyle and dietary choices can influence it, leading to Non-Communicable Diseases (NCDs) and affecting overall well-being. Participatory action research (PAR) is a promising approach to enhance communication and encourage individuals to adopt healthier behaviors and improve their health. In this study, we explored the feasibility of integrating the photovoice method with citizen science approaches to assess the impact of social and environmental factors on gut microbiota health. In this context, citizen science approaches entailed the involvement of participants in the collection of samples for subsequent analysis, specifically gut microbiome assessment via 16S rRNA gene sequencing. We recruited 70 volunteers and organized six photovoice groups based on age and educational background. Participants selected 64 photographs that represented the influence of daily habits on gut microbiota health and created four photovoice themes. Analysis of the gut microbiome using 16S rRNA gene sequencing identified 474 taxa, and in-depth microbial analysis revealed three clusters of people based on gut microbiome diversity and body mass index (BMI). Our findings indicate that participants enhanced their knowledge of gut microbiome health through PAR activities, and we found a correlation between lower microbial diversity, higher BMI, and better achievement of learning outcomes. Using PAR as a methodology is an effective way to increase citizens’ awareness and engagement in self-care, maintain healthy gut microbiota, and prevent NCD development. These interventions are particularly beneficial for individuals at higher risk of developing NCDs.

## Introduction

Participatory Action Research (PAR) is an appropriate strategy for approaching the population and understanding lifestyle and health decisions from the citizens’ perspective to build more successful interventions that promote healthier surroundings and convey challenging concepts.^1–3^ Within the field of PAR, the photovoice method has been gaining interest in social sciences,^4–6^ as well as in health care and disease management.^7,8^ It is defined as “a process by which people can identify, represent, and enhance their community through a specific photographic technique”.^9^ Citizen science initiatives, on the other hand, which encompass a variety of participatory models employing nonprofessionals as collaborators in scientific research, will enhance both citizen empowerment and biomedical research.^10,11^ These approaches can improve health communication related to the onset and progression of non-communicable diseases.

NCDs are not transmissible directly from one person to another, including diabetes, cancer, cardiovascular, and chronic respiratory diseases.^12^ Genetic factors and lifestyle habits like physical inactivity, unhealthy diet, and the harmful use of alcohol are tightly related to their development.^12^ The World Health Organization manifests that 70% of deaths globally (~41 million) are due to NCDs.^13^ Recent reports have shown that the gut microbiome may play a role in developing NCDs in conjunction with genetic and environmental factors.^14–16^

The gut microbiome comprises the genome of all the microorganisms that inhabit the gut; it plays a crucial role in the body’s nutritional, metabolic, physiological and immunological processes.^17,18^ shifts in host-microbiome interactions have a clinical impact on health. Gut microbiota dysbiosis, defined as the persistent imbalance of the gut microbial community, is associated with the onset of metabolic and inflammatory disorders, including non-communicable diseases (NCDs).^19,20^

There is great variability in terms of microbiome composition among different people.^21^ The environment has been demonstrated as a major driver of this variability, accounting for up to 20% of the variance in microbiome diversity.^22^ This high interindividual heterogeneity between people may be a source of differences in susceptibility to disease.^23^ Dietary and lifestyle choices play a significant role in the makeup and operation of the gut microbiota.^24,25^ Moreover, recent reports demonstrate an effect of social interactions in shaping the microbiome, which may play a role in microbiome-associated diseases.^26^ Hence strategies that might favorably modulate the gut microbiota to reduce the risk of many NCDs through diet and lifestyle changes are of great interest.^27^

Despite emerging evidence linking diet to host-microbiome interactions, there is little consideration of the gut microbiome in dietary recommendations.^28^ Public health researchers and practitioners are failing in their efforts to effectively communicate microbiome data, even though it is in high demand, particularly for promising microbiome-based therapies to maintain health and quality of life and prevent the development of NCDs.^29,30^ As a result, many citizens remain unaware of the implications of the gut microbiome in health and the development of NCDs.

Here we report on “#PictureYourMicrobes”, a participatory research initiative that brought together photovoice and self-tracking citizen science activities, with the primary objective of empowering citizens with risk factors for developing NCDs by raising awareness about the importance of taking care of the bacterial communities (microbiome) living in our bodies.

## Results

### Self-tracking citizen science tools helped to describe the population and characterize the participants’ gut microbiome

Up to 200 people showed interest in participating in the project; 70 volunteers that matched the inclusion criteria were selected through stratified random sampling. To obtain a profile involving each participant’s gut microbiome, diet, physical activity, and sociodemographic characteristics, we provided (a) self-reporting questionnaires to record data (see methods section) and (b) a gut-microbiome collection kit.

Table 1 describes participants’ sociodemographic characteristics obtained after analyzing the self-reporting questionnaires. Briefly, the participants ranged in age from 21–58 (mean age, 38), with 83% females and 17% males, and the majority (86%) had higher education. Most participants were based in the Madrid region (67%); however, people from seven regions of Spain also joined the project (Comunitat Valenciana, Cataluña, Galicia, Andalucía, Aragón, and Castilla la Mancha). We estimated the presence of risk factors for developing NCDs according to the World Health Organization^13^ and found that up to 67% of people had one or more risk factors for developing NCDs. Of them, 33% of the participants were overweight/obese, 20% of people smoked, 19.4% had a poor diet according to the Healthy Eating Index,^31^ 24% had low physical activity according to the GPAQ questionnaire,^32^ and 11.4% had other risk factors for developing NCDs (hypercholesterolemia, hypertension, asthma, high sugar blood levels).

**Table 1.** Participants’ demographics and nutritional parameters obtained after analysis of the food registry and GPAQ questionnaires.

Sociodemographic characteristics		*N* = 70^a^
Age (years)		38 (21–58)
Educational levelb	Secondary education	7(10%)
	Tertiary education	3(4.3%)
	Bachelor’s or equivalent	20(29%)
	Master o superior	40(57%)
Residence	Madrid region	47(67%)
	Other communities	23(33%)
Sex	Male	12(17%)
	Female	58(83%)
BMI (kg/m2	Normal range	47(67%)
	Overweight/Obese	23(33%)
Smoking		14(20%)
Physical activityc	Low	17(24%)
	Medium	23(33%)
	High	30(43%)
Healthy Eating Indexd	Poor	12(19.4%)
	Fair	15(24.1%)
	Good	18(29%)
	Very Good	12(19.4)
	Excellent	5(8.1%)
	NA	8

^a^mean (range); n(%). ^b^ According to the International Standard Classification of Education. ^c^ according to the GPAQ questionnaire.^35,d^ According to Healthy Eating Index.^37^

We performed microbial profiling from the stool samples collected by the participants by sequencing the 16S rRNA gene. After analysis, we found 7,685,850 high-quality sequences and identified 474 taxa. We calculated the relative abundance of the phylum present in each sample, the *Bacteroides/Firmicutes* ratio and the most common genera present. We developed an individualized report for each participant and discussed the microbial results in session 4. All the personal questions regarding the analysis were addressed to increase self-awareness about the gut microbiota and its relation to diet and lifestyle habits. We encouraged participants to use the results of their gut microbiome profiling and the food registries’ responses to enrich their photovoice narrative and to reflect in-depth on their habits.

### Photovoice resulted in four themes reflecting the concern of participants regarding gut microbiome health

Participants took 156 photographs during the entire Photovoice project and selected 64 that best reflected their food and lifestyle environment impacting gut microbiome health. A conceptual framework (Supplementary figure S2) was developed by researchers. It integrates the environmental, lifestyle, health-associated and diet-related factors that affect gut microbiome health. This framework helped participants group the photographs into four themes: Theme 1: “Balance”, referring to the importance of mental health and emotional well-being to achieve vibrant health. Theme 2 and 3: “Foodie” and “Mindful eating” related to the implication of diet in the modulation of the gut microbiome, emphasizing the socio-cultural aspect of food and the importance of eating consciously, including foods that help maintain intestinal health. Finally, theme 4: “Wellness”, including the physical activity routines performed by the participants aiming to boost health. Participants’ results are presented according to these themes, following SHOWED-based narratives and other group members’ related discussions.

#### Theme 1: balance

Leisure activities, having a pet, and promoting good healthy habits were pointed out as ways to have a balanced life that, according to our participants, is related to better gut microbiome health. Participants who worked emphasized that schedules and workloads often do not allow them to have good nutritional habits and were seeking balance. For example, a 50-year-old participant Supplementary Fig S3a: “A sedentary job has no long-term benefits to health” … “I feel additional stress, which has made my stomach not work the same, my body and my mood are resentful.” Also, participants emphasized the importance of leisure time to have better mental health and, therefore, better gut health. “Having time to dedicate to oneself is very important. It helps you to have a better mood and decrease stress and anxiety levels.” … “It is an important factor for good health, both mental and physical.” (Female, 30). In addition, having pets was suggested as an important factor for boosting well-being by helping, on the one hand, mental health and, on the other hand, influencing gut-microbiota, as an example Supplementary Fig S3b: “Pets bring happiness and company. In particular, regarding gut microbiome health, it has been proven that having pets, especially during the early years of life, when the microbiota is developing, promotes diversity and protects against immune-related diseases, such as allergies or asthma.” (Male, 22)

#### Theme 2: foodie

Participants noted that food is essential for communication within Spanish society, and plays a significant role in celebratory and family environments. These events usually happen in a relaxed atmosphere where healthy food is not prioritized. Participants gave relevance to moderate eating and finding the “healthier option” when celebrating and having leisure time with family and friends. Supplementary Fig 4a: “We tend to eat portions with great amounts of fat, salt, flours… much larger than when we eat at home.” … “I took this picture to show the other side of the coin when I have no time, or I don’t want to spend hours and hours preparing food.” (Female, 23). On the other hand, some participants suggest preparing the same dishes found in a restaurant with healthier ingredients and homemade. e.g., Supplementary Fig 4b: “A homemade dish from Chinese restaurants in Spain.”. “Rice is a very nutritious food containing resistant starch. Resistant starch is an excellent food for the microbiota as it becomes a prebiotic. It has a low glycemic index which reduces the inflammatory effects of the intestine” … “When I became more interested in leading a healthy lifestyle, I missed fast food dishes. Then I decided to make these meals at home in their healthy version.” (Female, 28)

#### Theme 3: mindful eating

Participants include photographs reflecting the importance of conscientiously taking care of the gut microbiome by including fiber, probiotics and a balanced diet. In Supplementary figure 5a, we showed a bowl of kefir. The participant who took this picture commented: “Our microbiota is made up of lots of living bacteria that take care of our intestinal health and prevent the development of diseases. When we take food with natural probiotics, we introduce living microorganisms that are very beneficial for our health and directly affect our microbiota.” (Female, 46). Moreover, incorporating fresh fruits and vegetables as sources of fiber was also deemed relevant. Supplementary Figure 5b: “The relationship between microbiota and food is very close, and local food can help us achieve a healthier, more sustainable diet to improve it.” … “Local foods have great virtues; the nutritional power of freshly cut fruit and vegetables, its texture, and the fact that it maintains all its properties and is ecological … unlike the food that we can find in any supermarket and/or shopping center.” (Female, 46).

#### Theme 4: wellness

Participants specified that exercise was essential for having better gut health. For many people, exercise is their way of disconnecting from their daily routine, work, and stress. Participants indicated that physical activity -such as going to the gym, engaging in outdoor activities, or doing indoor exercise- is closely linked to gut microbiota and overall well-being. In Supplementary figure 6a, a participant said: “Sport is essential to have a healthy life. Whether or not it is directly related to the microbiome, it has been shown to have a very beneficial impact; it reduces stress, improves sleep, increases our metabolism and muscle mass, densifies bone mass, and can be linked to a good relationship with food.” … “Physical exercise not only makes us feel good but also influences how we metabolize macronutrients healthily.” (Male, 26). Other participants also showed the importance of using alternative ways to commute to work, such as walking and using the bike, as reflected in the Global Physical Activity Questionnaire. “How we move around the city is a choice that affects both our health and the environment.” (Female, 35).“Moderate exercise outdoors, such as regularly commuting by bike, makes us more active and improves our physical condition and metabolism, including our microbiota.” (Male, 38). Overall, there was a consensus on the importance of creating a routine to exercise and improve health: Supplementary Fig 6b: “Regular physical activity can increase the growth of beneficial bacteria for our body” … “I try to exercise every day, doing yoga, doing static rowing or walking in the field.” (Female, 42).

### Learning outcomes related to gut microbiome health were accomplished after participatory action research activities

Learning was evaluated through the initial and final “knowledge questionnaires”. The percentage of correct answers before and after participation in the project is recorded in Supplementary Table S1. We define four learning outcomes: (i) State the microbiome concept. (ii) Distinguish between prebiotics and probiotic foods. (iii) Recognise the impact of nutrition on microbiome health. (iv) Discuss the implication of daily nutritional decisions affecting gut health. After project execution, participants could recall the microbiome concept, as seen in the increased percentage of correct answers in question 1 (Supplementary table S1). Moreover, they summarized different aspects of the microbiome, as seen in questions 7, 8 and 9 (Supplementary table S1). Some of the reflections after the focus group activities also showed the accomplishment of the learning outcomes. For example, a participant said: “I have learnt that we have many types of bacteria in the intestine, that each one regulates different things… but there are no more important bacteria than others… the most important thing is to have a balanced amount of each one of them”. Also, participants comprehend the difference between probiotics and prebiotics, as seen in the percentage of correct questions numbers 2, 3, 11 and 12 (Supplementary table S1). As seen in the focus group discussion reflections, participants understood and discussed the importance of daily nutritional decisions over gut microbiome health. A participant states: “The gut microbiome comprises a very diverse group of microorganisms with different metabolic functions, which are directly linked to our behavior and nutritional habits. We are what we eat”. Other participants said: “Diet and lifestyle have a great influence on the good condition of our microbiota… so it is never too late to make changes for improvement”, “I have learnt that a balanced microbiota is essential for physical and mental well-being. A diet that takes care of our microbiota is life insurance”.

### A detailed analysis of gut microbiome profiles revealed that participants with lower microbial diversity and higher BMI were linked to higher learning outcomes achievement

**Figure 1.**
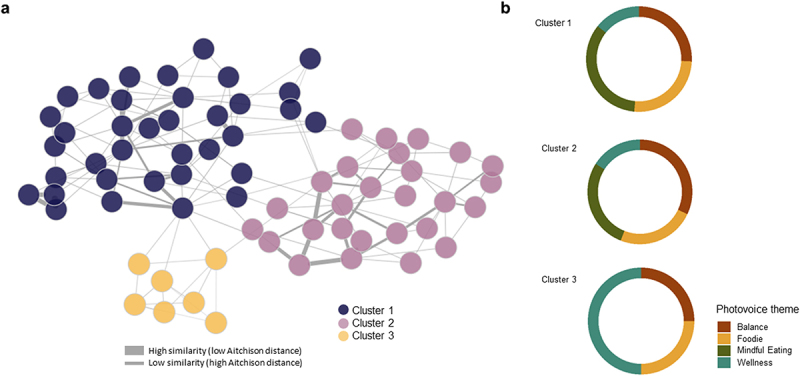
(a) Patient similarity network constructed from Aitchison distance and volunteers’ clusters, (b) Proportion of pictures provided to each photovoice category by participants belonging to each cluster.

Patient similarity networks are representations of patients grouped or categorized according to how similar they are to one another in terms of specific factors like genomic profiles.^33^ To construct a patient similarity network, we used the beta diversity of the microbiome calculated through the Aitchison distance^34^, in which each node is an individual participant, and an edge between participants corresponds to the similarity of their gut microbiomes measured by the Aitchison distance. After applying a hierarchical clustering algorithm, we identified three clusters of patients (Figure 1a). We analyzed participants’ sociodemographic characteristics in each cluster (Table 2). We found that Cluster 1 (*n* = 36) participants were characterized by higher weight and BMI than those from clusters 2 and 3. But we did not find differences according to eating habits or the Healthy eating index.

1a) Patient similarity network constructed with the Aitchison distance. Each node represents a patient, and the colors of the nodes indicate the clusters they belong to after applying k-nn clustering: cluster one (purple), cluster two (pink), and cluster three (yellow). Edges indicate the similarity between patients calculated using the Aitchison distance. The thicker the edge, the more similar the patients are. 1b) Proportions of photographs cataloged to each photovoice theme (Balance, Foodie, Mindful eating, Wellness) according to clusters.

**Table 2.** Participants’ demographics, lifestyle parameters and microbiome-knowledge results obtained after analysis of the food registry, GPAQ and ad-hoc microbiome-knowledge questionnaires according to the three clusters identified with patient similarity network.

Characteristic^a^		Cluster 1 (*n* = 36)	Cluster 2 (*n* = 27)	Cluster 3 (*n* = 7)	*P* value^g^
Age (years)		37	39	34	0.8
Gender	Men	8 (22%)	3 (11%)	1 (14%)	0.5
	Women	28 (78%)	24 (89%)	6 (86%)	
BMI (kg/m2)		25.3	23.3	21.2	0.060
Weight (kg)		69	63	56	0.038
F/B^b^ ratio		1.47	1.45	1.10	0.8
Physical activity^c^	Low	10 (28%)	6 (22%)	1 (14%)	0.9
	Medium	10 (28%)	10 (37%)	3 (43%)	
	High	16 (44%)	11 (41%)	3 (43%)	
Healthy eating index^d^	Poor	8(26%)	3(12%)	1(14%)	0.3
	Fair	7(23%)	6(25%)	2(29%)	
	Good	6(19%)	10(42%)	2(29%)	
	Very Good	5(16%)	5(21%)	2(29%)	
	Excellent	5(16%)	0(0%)	0(0%)	
Smoking		5(14%)	7(26%)	2(29%)	0.4
Two or more risk factors for NCD’s		13(36%)	7(26%)	2(29%)	0.8
Initial knowledge score^e^		13.1	13.2	14.6	0.012
Final knowledge score^e^		14	14	14.3	0.7
Variation in scoring^f^		0.89	0.83	−0.28	0.03

^a^mean; n(%). ^b^ Firmicutes/Bacteroidetes ratio. ^c^Physical activity according to the GPAQ questionnaire.^35 d^According to Healthy Eating Index.^37 e^Reported by the ad-hoc knowledge questionnaire. ^f^Variation in punctuation between final and initial knowledge questionnaire. ^g^Kruskall-Wallis rank sum test; Fisher’s exact test. In bold are shown those variables with a significant *p*-value.

**Figure 2.**
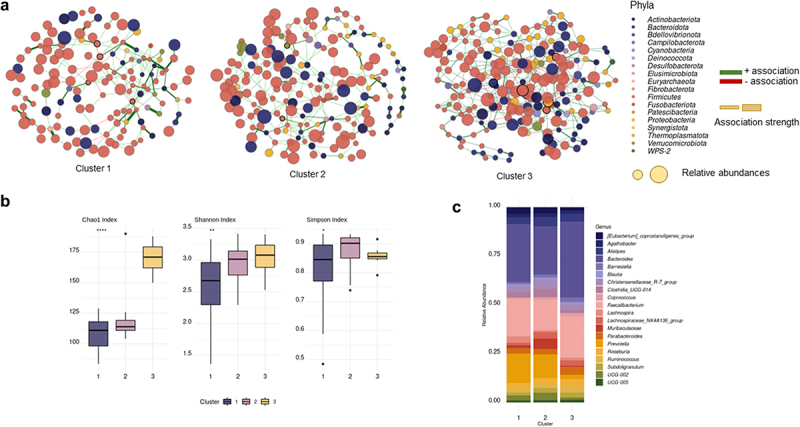
(a) Co-occurrence networks of the microbiome of each cluster of patients. (b) Alfa diversity indexes per cluster (c) Relative abundance of the top 20 genera according to each cluster.

We studied the microbial properties of each cluster’s participants by analyzing the microbiome’s alfa diversity and microbial abundance. We found that alfa diversity indexes from participants in cluster 1 were lower than participants from clusters 2 and 3 (Figure 2b); differences in the most abundant genera were also observed (Figure 2c). To investigate the microbial community structures of each patient sub-group, co-occurrence network analysis using Sparse InversE Covariance estimation for Ecological Association and Statistical Inference approach (SpiecEasi)^35^ was carried out (Figure 2a). In co-ocurrence networks, each node represents a microbial taxon, genus in our case, and the edges represent their association. If two microbes have a positive correlation, e.g., cooccur with each other, a positive association can be deduced, and a negative association can be deduced if the coefficient is negative, e.g., excludes the presence of each other.^36^ Table 3 presents the network properties. We found that network 3 showed a greater number of nodes and edges, as well as higher edge density values and connectivity between taxa (average degree), by contrast, the average network distance, referred to as the average path length, was the lowest. These results suggest that bacterial communities from participants of cluster 3 tended to be more connected to each other than the communities from clusters 1 and 2. We also determined keystone taxa, which are microbial taxa that are highly linked and exert a significant influence on microbiome structure and function regardless of their abundance. The keystone taxa found in the study varied according to the cluster (Table 3).

2a) Co-occurrence network of each cluster. The nodes in the graph represent different bacterial genera. The size of each node corresponds to its relative abundance, while the edges represent statistically significant associations between the nodes (*P* < 0.05). Green edges indicate positive relationships, while red edges indicate negative ones. The thickness of each edge indicates the strength of the association. Nodes highlighted in figure a correspond to keystone taxa calculated as nodes with betweenness and degree greater than Quantile 0.90. 2b) Alpha diversity indexes (Chao1, Shannon, and Simpson) from each cluster *** *P* < 0.001, ** *P* < 0.01, * *P* < 0.05.

**Table 3.** Network topological properties of microbial co-occurrence networks from each cluster.

Network properties	Cluster 1	Cluster 2	Cluster 3
Number of nodes	145	184	207
Number of edges	217	275	425
Average path length	4.011	4.55	3.21
Edge density	0.018	0.016	0.019
Average degree	1.49	1.49	2.05
Positive edge percentage	91.70	96	81.88
Keystone taxaa	*[Eubacterium] brachy* group *Candidatus Soleaferrea* *Lachnospiraceae* UCG-006 *Marvinbryantia*	*Pseudoflavonifractor* *Victivallis*	*Lachnospiraceae ND3007 group* *[Eubacterium] coprostanoligenes group* *Defluviitaleaceae UCG-011* *Brevundimonas* *Coprobacter* *UCG-004*

^a^Keystone taxa calculated as nodes with betweenness and degree greater than Quantile 0.90.

We identified the proportion of pictures provided to each photovoice category by participants belonging to each cluster to establish possible trends between groups (Figure 1b). We found that pictures provided by participants from Cluster 1 were mostly to food-related categories (Theme 1: Foodie, and Theme 3: Mindful Eating), whereas pictures from participants from cluster 3 were more related to non-food categories (Theme 2: Balance and Theme 4: Wellness).

Regarding the achievement of learning outcomes, we found that participants from Cluster 1 have the lowest initial scores in the initial knowledge questionnaires (13.1/15 points). However, after participating in the project, this group demonstrated the highest increase in score (+0.89 points, *P* < 0.05), followed by participants from cluster 2. Participants in Cluster 3 demonstrated a higher level of knowledge at the start of the project, and their scores remained consistent throughout (Table 2). This suggests that participants with less healthy microbiomes (Cluster 1) started with less knowledge about gut health, but improved their understanding through the project.

### Recommendations, dissemination of results and lessons learnt

Participants identified barriers and opportunities associated with each of the photovoice-defined themes. Supplementary table S2 shows the recommendations and measures extracted to solve the identified problems related to diet, lifestyle and the environmental and health-related factors affecting the gut microbiome health of the population and promote positive behaviors to achieve a healthy microbiota. The recommendations and the photographs selected by the participants and their narratives were published in an online photobook freely downloadable.^37^ with up to 236 downloads to the date (January 2023). We also organized a traveling exhibition at various civic centers to share our results, and it attracted more than 1,400 visitors.

**Figure 3.**
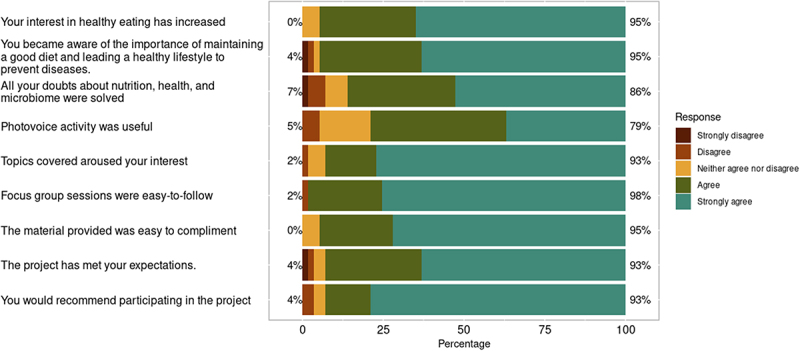
Results of the satisfaction and learning questionnaire presented on a Likert scale from 1 (strongly disagree) to 5 (strongly agree).

Finally, to evaluate to what extent participants have appreciated partaking in the project, we developed a questionnaire with a Likert scale regarding changes in habits, recommendations and global satisfaction after participation (Figure 3). We found that up to 95% of the participants were satisfied with their involvement in the project, and 93% would recommend participating. Up to 95% increased their interest in healthy eating, and 95% of the participants stated that they became more aware of the importance of maintaining a good diet and having healthy lifestyle habits to prevent diseases. Finally, 86% felt their questions about the nutrition-microbiome relationship were adequately addressed, and 79% found the photovoice project extremely beneficial.

## Discussion

Our work showed that integrating photovoice and citizen science is a novel way to understand how social and environmental factors affect gut microbiota health. Participants’ perceptions of their surroundings were revealed using photovoice. At the same time, self-tracking citizen science tools allowed us to assess their microbial profiles, quantify their physical activity levels, and better understand their nutritional choices. These data enabled participants to identify the strengths and weaknesses in their lifestyle habits, and create opportunities to improve their gut microbiome health.

Expert-driven science must improve in influencing people and communities.^30^ Considerable difficulties arise in communication between scientists and the general public, particularly in the context of microbiome research.First, it is challenging to stay on track due to the large volume of information generated, which makes it harder to choose accurate data for informing. Second, because editors hunt for clickbait news or significant information taken out of context, social media and the press may misrepresent some information. On the other side, researchers tend to discuss their work and focus on specific elements that are frequently difficult to understand. In contrast, even excellent science communicators often only provide a broad overview, typically based on reviews, without delving into specific subject details.^29^ On the other hand, from a scientific point of view, there are certain gaps in understanding how to focus microbiome research incorporating social, environmental, and behavioral factors that have been shown to have an impact on gut microbiome health.^38^ Participatory action research initiatives, which incorporate participants’ reflections and expectations into the transformation process, help simplify complex concepts, facilitate social change, and address the aforementioned issues.^30^

The activities developed in this study aimed to increase participants’ understanding of complex concepts related to the gut microbiome. By integrating various actions, we sought to promote critical awareness and prevent NCDs. The information provided to participants resulted from a combination of citizen science, critical pedagogy, and their active participation. A significant body of literature supports the use of photovoice for social sciences^13–15,32^, and the management of diseases such as diabetes.^16,17^ Our project explored the use of photovoice to reflect participants’ concerns regarding implication of lifestyle habits over gut microbiome and therefore holistic health. Discussion with the participants and their reflections during the project implementation revealed how they conceptualized the gut microbiome and the relation with the development of diseases. They agreed that certain lifestyle choices could negatively impact gut health, potentially accelerating the onset of NCDs. This comprehension of novel ideas, the implications of one’s control over one’s health, and the prevention of diseases through maintaining the health of the microbiome are in line with the theoretical underpinnings of photovoice’s work on critical consciousness, which holds that advocacy for change can be accomplished through a critical analysis of a given subject and a process of self-awareness.^9^

Gathering data and profiling the gut microbiome helped us tailor the information provided to each participant, enabling more insightful reflections. Evidence from educational projects suggests that the analysis of their own microbiome data enhances engagement, interest, and perceived learning experience in students from different courses.^39,40^ We demonstrate that understanding their own microbiome data allows participants to raise awareness about the importance of taking care of their gut health.

In addition to the educational role of our project, we were also able to study in depth how specific environmental factors such as diet, physical activity, and stress impact the gut microbiome of a cohort of volunteers. We use a personalized medicine tool, the patient similarity networks, to create subgroups of patients based on the similarities of their gut microbiome.

Patient similarity networks have proven to be a helpful tool for precision medicine, notably for clustering patients.^41,42^, diagnosis, and prognosis.^43^ Subdividing patients into homogenous subgroups based on biology, disease progression, and response to treatment serves as the foundation for the patient similarity network paradigm, allowing accurate, generalizable classifiers that can integrate diverse data and easily manage missing information. Prior research has shown the value of patient similarity networks to identify labels or subtypes in patients with different diseases based on homogeneous signatures.^33^ We employed the Aitchison distance – a measure of microbial beta diversity- to construct a network which allowed us to integrate the microbiome status with the cohort’s phenotype despite its apparent homogeneity, allowing us to describe three subgroups of patients. The established clusters allow the identification of persons with a gut microbiome that is more prone to obesity or with anti-obesity bacteria. Finding that patients with a less diverse microbiome are more likely to be overweight or obese and that their photovoice reflections on the project were more focused on food and diet-related issues, but that they also benefited the most from taking part in the project because they learned more, indicating that those at risk of obesity or NCD benefit more from these kinds of interventions.

The study of microbial composition from an ecological perspective allows the detection of changes in the microbiome that may be hindered when using conventional statistical analytical techniques. We were able to identify specific microbial genera as the pillars of the microbial community by using co-occurrence networks to examine the microbial connection of the microbiome. The keystone taxa are described as microbiome structure and function drivers, particularly their interaction network, which plays an essential role in microbial functions and disease progression.^44^ We found that keystone taxa from the network of Cluster 1 patients was associated with high-fat diet^45^ (*Lachnospiraceae* UCG-006) and higher BMI^46^ (*Marvinbryantia* spp). Conversely, keystone taxa found in Cluster 2 and 3 were associated with weight loss^47^ (*Pseudoflavonifractor spp*.), lipid metabolism^48^ ([*Eubacterium*] *coprostanoligenes* group, *Defluviitaleaceae* UCG-011), and short-chain fatty acid production^49^ (*Lachnospiraceae* ND3007 group, UCG-004).

Regarding limitations, participatory action research presents logistical challenges in recruitment, coordinating meetings and collecting and managing personal data. These were mitigated to the best of our ability by working online and having flexibility in terms of scheduling focus group reunions. Our recruitment strategy highly based on social media made our population with some characteristics -including the age range and the high proportion of women- that may limit the data presented in this project. As well, the participants in this study were limited by lacking racial and ethnic diversity as the majority of participants were white with a higher degree of education, we acknowledge that health is further complicated by race and socioeconomic status, and future research should aim to explore these topics through a more expanded view.

## Materials and methods

This participatory action research project was conducted from April to September 2021.

### Participants recruitment

A purposive sampling strategy was used to engage participants.^50^ The recruitment strategy was firmly based on social media, using Facebook, Instagram, and Twitter channels from networks specialized in disseminating content related to nutrition and health. The project was also publicized on the platform: “Citizen Science Observatory” (https://ciencia-ciudadana.es/). Inclusion criteria were: 1) ages between 18–65 years, 2) live in Spain, 3) speak Spanish, 4) had no impediment to managing a digital camera, 5) not having a psychiatric disease or alimentary disorders, and 6) agreed to attend four group sessions for two months. Exclusion criteria were: 1) psychiatric illness or eating disorder, 2) pregnancy or lactation, 3) people without access to telephone or internet, 4) low level of communication in Spanish, and 5) people with a disability or impediment that does not allow accurate monitoring of activities. Study size was calculated with the pwr package Version 1.3 of Software R^51^ assuming a change effect of 2 points as described in other works^52^ with a standard deviation of 5, an error alpha of 0.05 and a power of 0.8, taking into account a dropout of 25% and 10% of poorly completed questionnaires.

### Ethical aspects and data processing

Protocols and methodology used in the present study comply with the ethical principles for research involving human subjects laid down in the Declaration of Helsinki (1964) and its modifications. The study was approved by the Research Ethics Committee of the IMDEA Food Institute (PI-047; Approval date: March 11th, 2021). Participants were informed in detail about the different stages of the project both orally and in writing. The researchers collected signed informed consent prior to the first evaluation. This document included specific consent to microbial profiling. Data compiled during the study was processed by applying dissociation criteria, making the volunteers’ data anonymous, in compliance with the current Spanish legislation (Organic Law 15/1999 of December 13th, on the protection of Personal Data).

### Photovoice procedure and focus group sessions

Six Photovoice groups were created, homogeneous in age, educational level, and geographic region. Each group met online for four sessions, held every two weeks, and lasted approximately one and a half hours. Co-facilitators (an academic-based researcher and a nutritionist) facilitated the Photovoice groups, intervening only to explain the objectives of each session, involve everyone, and ensure that each participant was partaking. Each group session was recorded and transcribed. Sessions were divided into an initial group meeting, small group discussion sessions, and a final meeting.

In supplementary figure S1 is shown a scheme for the organization of the sessions. Briefly, in the first session, the research team introduced the project and the photovoice methodology. Participants performed an “initial knowledge questionnaire” (see methods section 2.4.1), and co-facilitators explained the procedure to collect stool samples for microbial profiling and provided explanations on how to fill the questionnaires and self-reporting data (see methods section 2.4.1). In session 2, co-facilitators discussed the relevance of nutrition and daily routines on gut microbiota health, and participants were invited to take 2–3 images that appropriately represented the influence of their lifestyle on microbiome composition. Participants were also given a template with an adapted version of the SHOWED mnemonic method to help guide discussions about why they shot the photograph and what it meant to them.^9^ The template included the following questions: 1) What does the photograph show?; 2) What is really going on here? 3) How does this relate to our lives and the health of the microbiome? 4) Why did you take this photograph? 5) What can we do about it? In the same session, participants received photography training. In session 3, participants discussed their photographs following SHOWED methodology and grouped them into themes considering the conceptual framework of the project developed by researchers (Supplementary figure S2). In session 4, participants received their microbial profiling results and discussed the results considering the photographs and joining with their reflection after filling out the questionnaires and self-reporting data. Finally, participants created policy recommendations to improve their diet and lifestyle habits to improve their microbiome health and completed the “final knowledge questionnaire”.

### Self-tracking citizen science tools

#### Questionnaires and self-reporting data

Different questionnaires were provided asking for microbiome knowledge, lifestyle habits, satisfaction and learning data. a) Microbiome knowledge questionnaire: An ad-hoc 15-item knowledge questionnaire about the role of the microbiome in the development of non-communicable diseases and their relationship with healthy habits was developed (See Supplementary table S1). Each participant’s knowledge score was computed by assigning 1 point to each correct response or 0 points, generating a 0 − 15. Higher scores represented a greater level of microbiome knowledge. The ad-hoc knowledge questionnaire was presented in session one before the project’s development: “Initial knowledge questionnaire”, and in session four after executing all the activities: “Final knowledge questionnaire”. b) Lifestyle questionnaires: A validated food record of 72 h consumption^53^ in which participants had to sum up all food and drinks ingested during three days (2 weekdays and 1 Sunday or holiday) was delivered. Furthermore, the validated Global Questionnaire on Physical Activity of the World Health Organization was applied to assess the degree and intensity of physical activity.^32^ c) Satisfaction and learning questionnaire: an ad-hoc satisfaction questionnaire was also developed to evaluate to what extent participants engaged in the project. All items of the “satisfaction questionnaire” used a 5-point Likert response format (1 = strongly disagree to 5 = strongly agree for beliefs, and 1 = never to 5 = very often for attitudes).

#### Microbial profiling

Stool samples were collected in the OMNIgene•GUT collecting kit (DNA Genotek, Ontario, Canada) and sent by postal service in the next two days after sampling for microbial profiling by 16S rRNA gene sequencing. DNA was extracted using the QIAamp DNA stool Minikit according to the manufacturer’s instructions (QIAGEN, Hilden, Germany). The variable V3 and V4 regions of the bacterial 16S ribosomal RNA gene (16S rRNA) were amplified from the fecal DNA and sequenced with the Illumina MiSeq platform (2 × 300).

### Data analysis

Descriptive results are presented as the mean, interquartile range. Statistical analyses were performed using R software.^54^ Qualitative and quantitative analysis of the diet was performed using the DIAL nutritional software v 3.15 (Alce Ingenieria, Madrid, Spain), and the calculated Healthy Eating index.^31^ was delivered to participants. Likert questionnaires were analyzed using the Likert package in R software.^55^ Statistical analysis was performed with the Kruskal-Wallis, Fischer exact test or an independent Student’s t-test accordingly to data, being *P* < 0.05 values considered statistically significant.

For the microbial profiling, data were processed using the Quantitative Insights Into Microbial Ecology program (QIIME2) version 2022.2.0.^56^ and annotated with the SILVA v.132 16S rRNA gene reference database.^57^ The relative abundance of each Amplicon Sequence Variant (ASV) and alpha diversity (Shannon, Chao1, and Simpson indexes) were calculated using the phyloseq R package.^58^ A final report was provided for all participants showing the main phyla, the *Firmicutes/Bacteroidetes* ratio, and the top 10 genera present in the sample.

#### Further microbiome analysis

A similarity network was constructed with netComi package^59^ using Aitchison distance,^34^ measuring the Beta diversity of the microbiome. Nodes represented each patient, and the edges the dissimilarity value, edges connecting nodes were selected by constructing a k-nearest neighbor graph. Clustering was performed with the hierarchical algorithm based on dissimilarity values. Microbial co-occurrence networks were constructed using Sparse InversE Covariance estimation for Ecological Association and Statistical Inference (SpiecEasi) method^35^ with netComi package.^59^ Nodes represented the microbial genus and edge the co-occurrence. Edges connecting nodes were selected by Student’s t-test, and only edges with a significance level after multiple testing adjustment greater than 0.05 were selected. The thickness of each edge indicates the strength of the association. Keystone taxa were defined as highly connected taxa that significantly influenced microbiome structure and function, regardless of their abundance^44^ and were determined as hubs with degree and betweenness greater than the 90 quartiles.

## Supplementary Material

Supplemental Material

## Data Availability

Sequence files for all samples used in this study have been deposited in ENA under the accession number PRJEB59312. Metadata have been deposited in Figshare (https://doi.org/10.6084/m9.figshare.21977198.v1). A full record of all data analysis and original R scripts are available in GitHub (https://github.com/laurichi13/PYM).
