# Supplementary material for: Citizen science helps to raise awareness about gut microbiome health in people at risk of developing non-communicable diseases
Source: Gut Microbes. 2023 Aug 2;15(1):2241207. doi: 10.1080/19490976.2023.2241207 (PMC10399471; doi:10.1080/19490976.2023.2241207)

**Supplementary table 1.** Questions of the ad-hoc microbiome-knowledge questionnaire and percentage of correct answers after and before participation in the project.

| **Question** | **Before participation** | **After**  **participation** |
| --- | --- | --- |
| 1. Do you know what the microbiota is? | 89% | 100% |
| 2. Are probiotics foods or supplements that contain live microorganisms that promote benefits in the body? | 98% | 98% |
| 3. Are prebiotics foods or supplements that contain inactivated microorganisms that promote benefits in the body? | 47% | 58% |
| 4. Can a person's microbiota be modified? | 96% | 100% |
| 5. Can the microbiota influence the development/appearance of certain diseases? | 100% | 100% |
| 6. Can diet alter the microbiota? | 100% | 100% |
| 7. Can you tell what microbiota a person has? | 100% | 100% |
| 8. Is there only microbiota in the intestines? | 91% | 93% |
| 9. Do you know what a microbiota transplant is? | 60% | 77% |
| 10. Antibiotics can modify the microbiota? | 100% | 100% |
| 11. Do you know which foods are probiotics? | 68% | 95% |
| 12. Do you know which foods are rich in fiber? | 96% | 98% |

**Supplementary table 2.** Recommendations and interventions developed by participants after the photovoice project execution. All recommendations are shown according to the photovoice theme.

| **Recommendation** | **Interventions** | **Theme** |
| --- | --- | --- |
| - **Increase the quality of leisure time moments**   To reduce stress caused by the high pace of daily life, work, and lack of rest. | - Socialising to maintain an optimal mood and keep the brain active. - Getting 8 hours of sleep. - Going for a walk in a pleasant environment. | *Balance* |
| - **Don't give up the food we like**   To maintain good social health by avoiding daily monotony. | - Eating out with family and friends. - Consuming "unhealthy" foods occasionally. - Cooking with healthier ingredients and at home. | *Foodie* |
| - **Implement nutritional education programs**   So that the new generations become aware of the importance of a healthy diet in their microbiota. | - Providing nutrition education classes in schools. - Teaching the smallest healthy eating habits in the family environment. | *Mindful Eating* |
| - **Increased availability of fresh fruits and vegetables**   To achieve a more varied and healthy diet by taking advantage of the seasonality of food. | - Choosing zero-kilometer products in local internet stores. - Creating organic gardens at home. - Replacing prepared or ultra-processed foods with fresh products. | *Mindful Eating* |
| - **Increase consumption of prebiotics and probiotics**   To improve and strengthen the health of our microbiota. | - Making at home Kefir, Kimchi, or Kombucha. - Attending prebiotic food courses. | *Mindful Eating* |
| - **Encouraging non-motorised means of transport**   To increase daily physical activity. | - Promoting movement on foot. - Making use of the cycling in urban areas. | *Wellness* |
| - **Promote sport**   For the sedentary, motivating citizens to practice collective or individual exercise. | - Performing different exercises at home with the necessary equipment. - Attending classes or scheduled activities. | *Wellness* |

**Supplementary Figure 1.** Schematic organisation of participatory action research activities developed within the study.

**
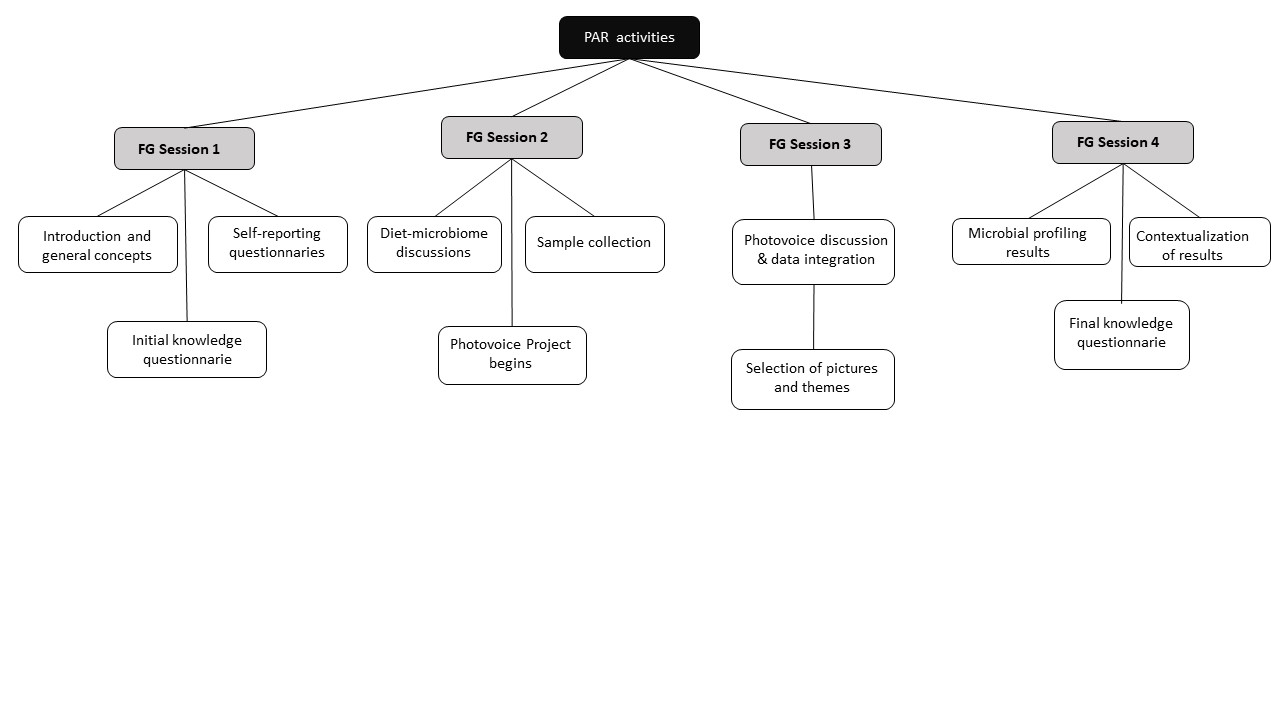
**

PAR: Participatory Action Research, FG: Focus group

**Supplementary Figure 2.** Conceptual framework of the photovoice project.


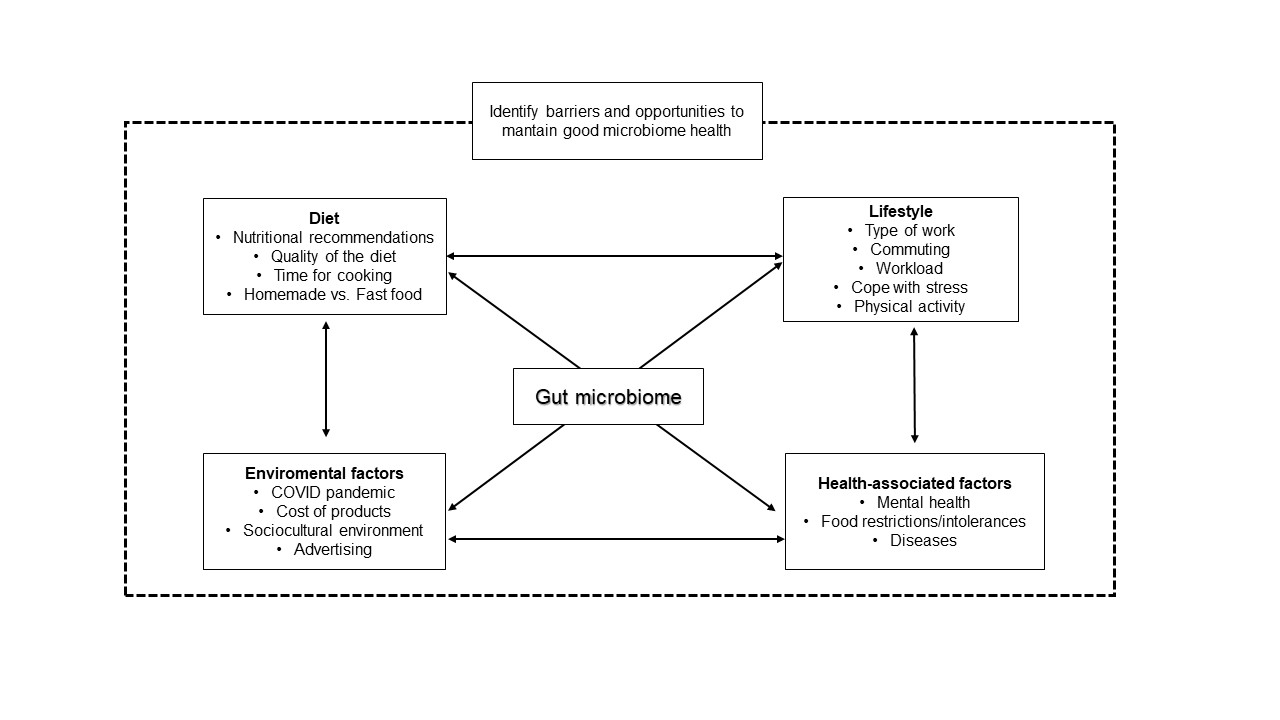


**Supplementary figure 3. (a)** Photograph reflecting stress and high workload: "Working from home, many hours of sedentary lifestyle and loneliness" Theme: Balance. **(b)** Photograph reflecting the importance of having pets to wellness: "The implication of having a pet with my microbiota". Theme: Balance.


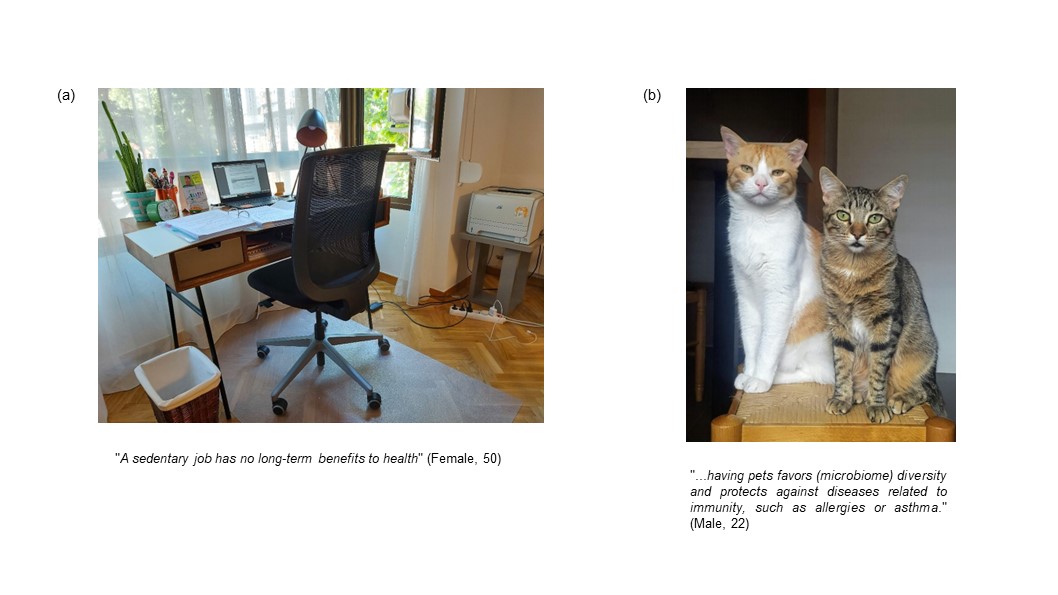


**Supplementary figure 4. (a)** Photograph reflecting unhealthy food elections: "Fast food, to calm hunger quickly and easily" Theme: Foodie. **(b)** Photograph reflecting the importance of better health decisions in leisure time: "Bowl with Asian style rice". Theme: Foodie.


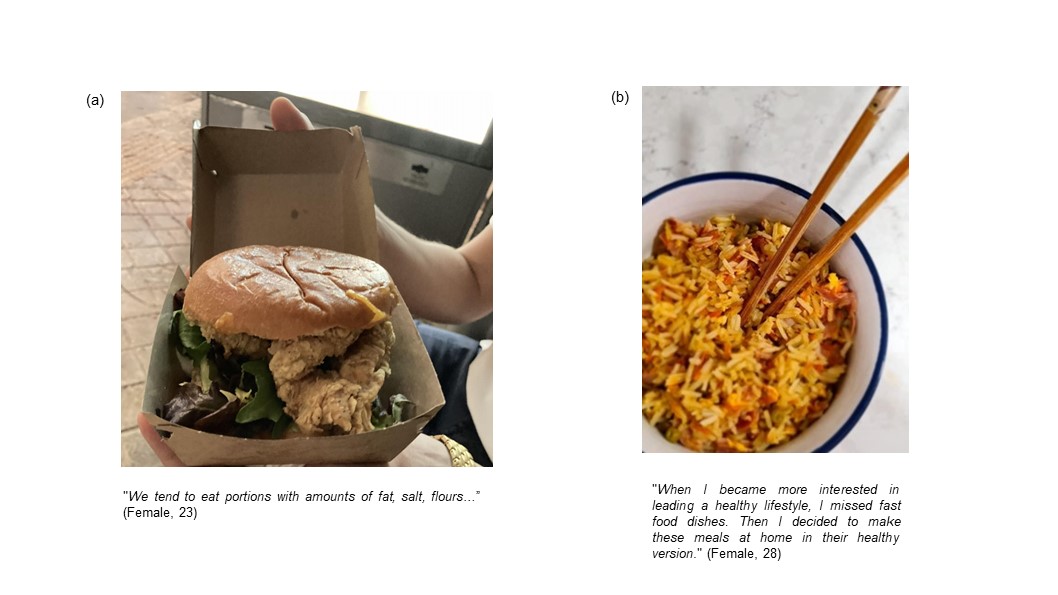


**Supplementary figure 5. (a)** Photograph of homemade probiotic food: "The superfoods are the ideal choice" Theme: Mindful eating (b) Photograph showing fruits and vegetables: "Local farmers who produce high-quality food". Theme Mindful eating.


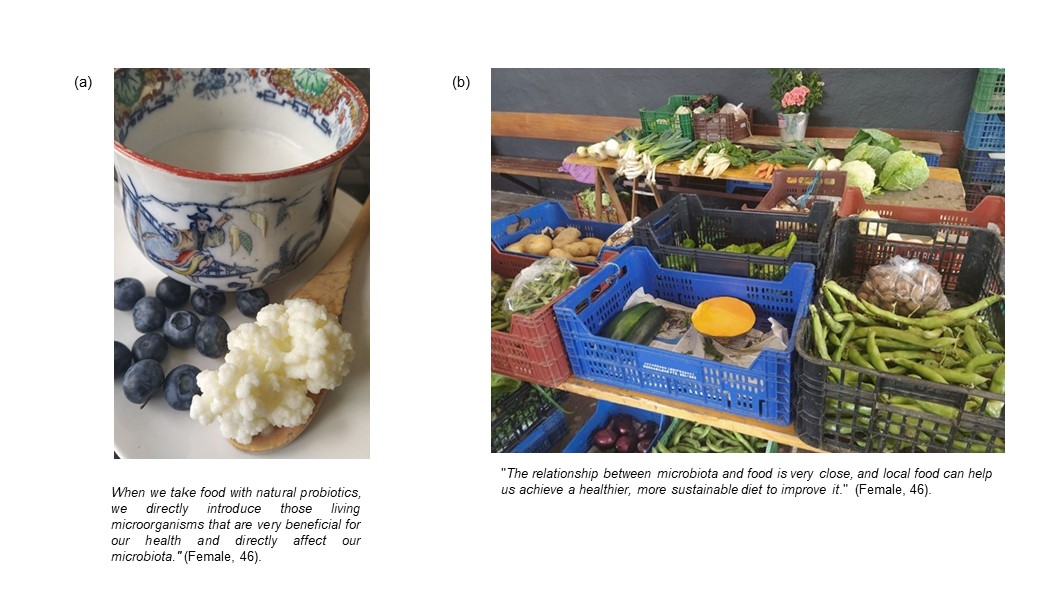


**Supplementary figure 6. (a)** Photograph reflecting an improvised homemade gym: "Gym at home, in an easy, practical and comfortable way" Theme: Wellness. (b) Photograph reflecting the benefits of doing exercise: "Yoga posture in a pleasant environment". Theme: Wellness.


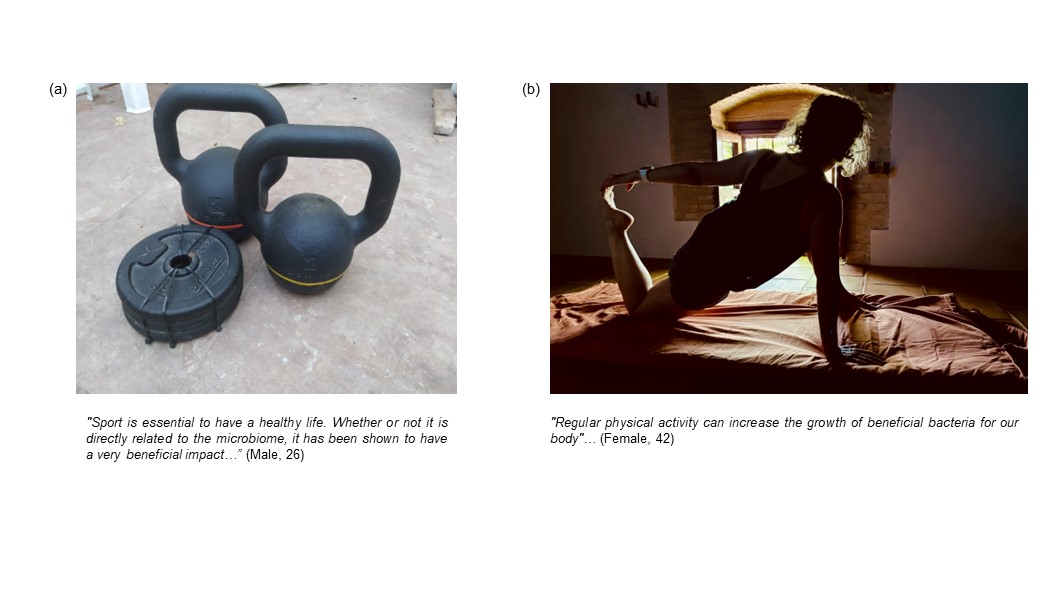

Supplement: Supplemental Material [file KGMI_A_2241207_SM5921.docx]
